# Muscle Mass as a Biomarker for Health Status and Function in Pediatric Individuals with Neuromuscular Disabilities: A Systematic Review

**DOI:** 10.3390/children11070815

**Published:** 2024-07-03

**Authors:** Isabella R. Ferrara, Cristina L. Sadowsky

**Affiliations:** 1 International Center for Spinal Cord Injury, Kennedy Krieger Institute, Baltimore, MD 21205, USA; ferrara@kennedykrieger.org; 2 Department of Physical Medicine and Rehabilitation, Johns Hopkins School of Medicine, Baltimore, MD 21287, USA

**Keywords:** muscle mass, neuromuscular disorder, functional ability, bone health, cardiometabolic risk

## Abstract

This systematic review aims to investigate the relationship between muscle mass and specific health outcomes in pediatric populations with neuromuscular disorders. A search was performed for any relevant studies published in English from 1996 to 2023 in five databases. To be included in this analysis, articles must have had participants with an average age ≤21, focus on children with neuromuscular disabilities, and primarily examine relationships between muscle mass and any functional or health outcomes measure. Studies including typically developing children were used to contrast and enhance findings. Thirty-two studies were included, with 10,129 unique individuals represented: seventeen studies focused on healthy/typically developing children; seven on children with cerebral palsy; three on children with Duchenne muscular dystrophy; two on children with sarcopenia; and one study each on children with osteoporosis, congenital muscular dystrophy, and other various neurologic disorders. Thirteen studies assessed functional outcomes, ten assessed bone outcomes, and nine assessed other cardiovascular/metabolic outcomes. All of the included studies demonstrated relationships between muscle mass and respective outcomes in varying measures. The results of this review demonstrate that there is a consistently recognized relationship between muscle mass and important health outcomes in children, supporting clinically targeting muscle mass as a means to optimize desired outcomes.

## 1. Introduction

Sarcopenia, defined as loss of skeletal muscle mass (SMM), is mostly studied in able-body aging adults, where it is recognized as a pathologic condition. The European Working Group on Sarcopenia in Older People defines age-related sarcopenia as muscle failure based on three criteria: (i) muscle strength, (ii) muscle quality/quantity, and (iii) physical performance [1]. Thus, sarcopenia is strongly associated with loss of physiological and day-to-day function [2]. Sarcopenic obesity is a related condition characterized by low muscle mass associated with high body fat mass [3]. 

But not only aging adults lose muscle mass; adults and children with neurologic conditions affecting the neuromuscular systems, like cerebral palsy (CP), muscle dystrophies, and other forms of paralysis, also have decreased muscle mass related to neurologic deficit, decreased mobility, and intrinsic muscle pathology. Taken together, neuromuscular disorders are a group of conditions that can impact muscle function either through specific muscles or the consequences of peripheral nervous-system pathologies [4]. When examined individually, these conditions are rare, with prevalence rates lower than 50/100,000 worldwide. As a collective, however, neuromuscular disorders have similar prevalence rates to Parkinson’s disease, ranging from 100 to 300/100,000 worldwide [5]. CP, one of the most frequent conditions impacting the neuromuscular and musculoskeletal systems, affects one’s ability to control his or her muscles [6]. This disorder affects 1–4 children per 1000 live births in the world [7], totaling from 140,000 to 560,000 cases per year. Duchenne muscular dystrophy (DMD) is another rare diagnosis causing a decrease in the production of the protein dystrophin; it affects 1 in 5000-to-6000 people assigned male at birth, with a prevalence rate estimated to be 10 cases per 100,000 males [8]. Congenital muscular dystrophy (CMD) is a similar condition, with prevalence rates estimated at 1–9 per 100,000 [9]. Some of the most common side effects of neuromuscular diseases include muscle weakness and muscle mass loss [6,8,9] because of damage to the nerves in the peripheral nervous system. Like the sarcopenia of aging, this earlier onset of sarcopenia and sarcopenic obesity can result in impaired physiologic and functional performance, leading to adverse health-related outcomes, including deficits in cardiometabolic function, mobility, and bone health. Patients with neuromuscular disorders often have reduced functional mobility, increased risk of bone injury/fracture, and nutritional issues arising from various swallowing- and feeding-disorder comorbidities, with profound effects of quality of life [10].

Muscle mass (MM) is defined as the amount of muscle in the body and includes skeletal muscles, smooth muscles, and cardiac muscles [11]. SMM is commonly described in studies and clinical practice using terminology such as fat-free mass (FFM) and lean body mass/tissue (LBM). A detailed description of these parameters is outlined in Table 1. As more accurate MM measurement methods have become available, there has been a shift away from traditional measures of body weight and body mass index (BMI) as indicators for future pathology. This shift results from ever-evolving technology that allows clinicians to take more precise measurements of body composition. Techniques such as Dual X-Ray Absorptiometry (DXA), magnetic resonance imaging (MRI), bioelectric impedance (BIA), and computed tomography (CT) are the objective methods used to collect body composition data, including MM, fat mass, bone mass, and bone density measurements [12]. DXA is emerging as the most clinically accessible tool and has a strong correlation with the bioelectrical impedance analysis (BIA) [13]. Of course, the DXA-based measurement of MM can be confounded by hydration levels in soft tissue, which can vary across sex, age, and presence of chronic disease [14]. Like MRI, it is center-based and subject to body size limitation, and variations between manufacturers’ software do not allow for comparisons between different scans. CT scans carry the additional risk of exposure to radiation. Ultrasound (US) has also been used to estimate MM but less commonly, because of operator-skill dependency, and even more soft tissue hydration errors [15]. Older MM assessment methods, like body weight and BMI, are outdated and do not accurately describe the variability in body composition [16]. Consequently, these methods can overlook associations between abnormalities in specific body tissues and health risks. In contrast, newer MM assessment tools provide more objective ways to predict functional performance, like mobility, strength, balance, and gait; bone outcomes like bone mineral content (BMC) and bone mineral density (BMD); and cardiometabolic clinical outcomes [17]. Therefore, MM becomes an increasingly important health outcome that should be understood and could be more frequently utilized as a target of intervention in order to make objective therapeutic decisions—especially for individuals with neuromuscular disorders.

While there are limited studies on effects of sarcopenia and sarcopenic obesity in children, there are some that associate loss of MM with a higher metabolic risk based on the presence of other identified risk factors, including increased waist circumference (WC), elevated blood pressure (BP), low/high-density lipoprotein cholesterol, hypertriglyceridemia, and glucose impairment [3]. The metabolic risk appears to increase proportional to the degree of MM loss [19]. This relationship is maintained throughout adulthood, where sarcopenia is associated with both a higher risk of metabolic syndrome [20] and poorer cardiopulmonary performance and disease [2]. 

There is extensive research on sarcopenia’s connection to bone health and bone strength in both children and adults. In pediatric populations, lean body mass (LBM) is a significant predictor of bone mineral content (BMC) [21]. In addition, optimal MM development throughout childhood helps prevent the development of osteoporosis and sarcopenia in adulthood [19]. Further research in adults confirms this relationship by demonstrating that low MM increases the risk of low bone mass and osteoporosis [22]. These findings strongly support the role of MM in the development of pathologic bone density losses [23].

There is a smaller body of literature related to sarcopenia’s effect on functional mobility in pediatric populations. Some studies demonstrate that children with low MM walk significantly shorter distances during the 6 min walk test and exhibit longer times for the timed up-and-go tests [24]. In younger adults, the degree of sarcopenia is associated with lower physical activity and decreased strength, particularly on measures of physical performance, like stair-climb tests, walking tests, and handgrip-strength assessment [25]. Similarly, adults with low MM demonstrate decreased physical capabilities, a higher rate of mobility disorders, increased chances of falls/fractures, and lower degrees of activities of daily living (ADL) completion [2]; these effects are worsened with advancing age [26].

All of these findings point to the role that MM can play in multiple health outcomes, lifelong. 

While clinicians recognize the importance of MM as a measurement tool in a wide array of pathologies, most of the existing research focuses on studying MM in aging adults or the typically developing population, particularly elite-level athletes. Given the gaps in the literature, particularly for a pediatric population with immobility related to neuromuscular disorders, examining MM’s connection to health-related outcomes appears worthwhile and could lead to the adoption of MM as an objective, clinically relevant surrogate measure that can be targeted for intervention in order to minimize comorbid pathology and further loss of function. Children with neuromuscular disabilities are especially underrepresented in research yet experience many of these adverse health outcomes. This paper aims to provide a comprehensive, systematic review that presents data regarding MM and its correlations to specific outcomes in pediatric population with selected neuromuscular pathologies (i.e., CP, DMD, and CMD), specifically looking at functional/mobility outcomes, bone health, and cardiometabolic-related outcomes. We also aimed to evaluate some of the differences in findings for typically developing and differently abled children. Lastly, this paper emphasizes how MM can be used objectively as a biomarker for clinically relevant outcomes. 

## 2. Materials and Methods

Using the Preferred Reporting Items for Systematic Reviews and Meta-Analyses (PRISMA) [27] as a guide, a systematic review was performed. See Table S1 for the detailed PRISMA 2020 checklist [28]. A research protocol was used to outline the review process, including the search method and databases to be used; relevant search terms; and population, intervention, comparison, and outcome (PICO) details. Inclusion and exclusion criteria were defined before the review process.

### 2.1. Literature Search

Five databases were used for the literature search: Web of Science, PubMed, CINAHL, Cochrane, and EMBASE. Multiple search strategies were employed, focusing on MM measurements within the pediatric population to determine associations between MM and functional outcomes for rehabilitation and recovery. Search terms were used in different combinations and in conjunction with Boolean phrases (and/or) to find articles. Table 2 demonstrates how terms and Boolean phrases were applied. Filters such as “studies involving humans”, “published in English”, and “access to full text” also restricted search results. Beyond a literature search, reference lists of included sources were scanned for any relevant studies missed. All search results were uploaded into Covidence, an online platform to streamline systematic and other reviews [29].

### 2.2. Study Selection

Duplicates were removed automatically, using Covidence online software (https://www.covidence.org, accessed on 22 November 2022), and then manually screened by one reviewer to ensure accuracy. After this process, the following inclusion criteria were discussed by both authors and used to screen titles and abstracts to assess article relevance:

1. Average participant age is ≤21 years; studies with individual participants over the age of 21 were included as long as the average age remained below. This was to ensure that studies represented individuals across all stages of puberty and post-puberty; only one study [30] had participants over 21 (range was 18–22 years old, with average age of 19);
2. The study focused on individuals with neuromuscular disabilities; studies with typically developing children were included to help emphasize differences between children with neuromuscular disabilities and those without;
3. Primary examination included assessment of relationship(s) between MM (measured by LBM or FFM) and functional outcome measures, including but not limited to gait, mobility, bone, cardiovascular health, and metabolic health.

Following the title and abstract screening, one reviewer conducted a full-text review to assess eligibility. All study and publication types were included except for other reviews, conference proceedings, or poster presentations. No parameters were set regarding sample size, year of publication, country of publication, or intervention. Studies were excluded from analysis if (i) confounding factors were present (i.e., lean/muscle mass was not isolated from fat mass in measurement), (ii) mass was measured with a non-recognized/accepted method (DXA, MRI, BIA, and CT), (iii) studies examined adult population (iv) outcomes were not relevant to the aim of this review or (v) articles were inaccessible to the reviewer. The second reviewer independently read and confirmed the articles finally included in the paper. Any disagreements were resolved by consensus. 

### 2.3. Data Synthesis

One reviewer extracted the following data from each study included for review: author(s), year of publication, country of origin, study design, sample size, population description (i.e., age, gender, and condition), outcome measures and conclusions. Relevant data were pooled and summarized in the table provided below with four major sections: (1) Study Information; (2) Sample Characteristics and Measurements; (3) Outcome Measures; and (4) Conclusions.

## 3. Results

### 3.1. Study Screening and Inclusion

**Figure 1:**
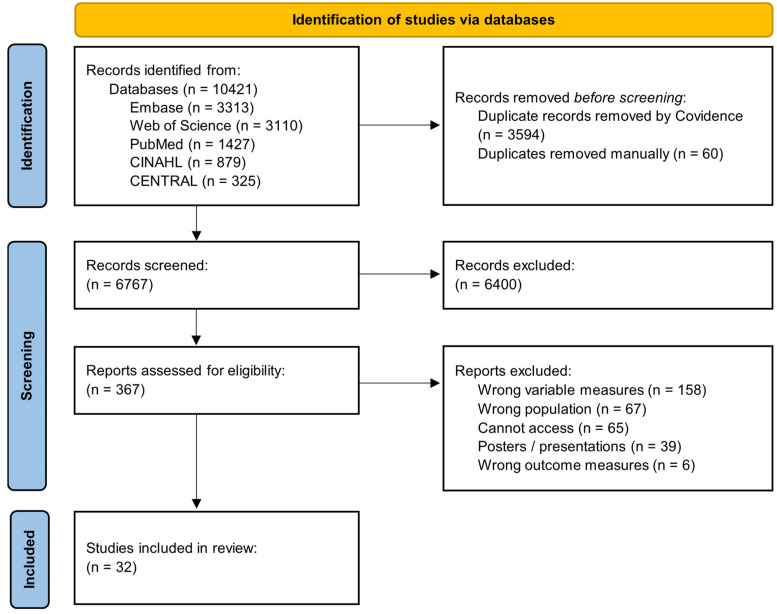
PRISMA flowchart.

The PRISMA flowchart depicting the literature search results is depicted in Figure 1. Searches identified a total of 10,421 articles across five databases, 3654 of which were duplicates and removed. The remaining 6767 were screened based on title and abstract, 6400 were deemed irrelevant. After screening, a total of 367 articles underwent a full-text review based on the inclusion and exclusion criteria: 158 used incorrect variable measures, 67 focused on different than intended population, 65 could not be accessed, 39 were posters or presentations, and 6 used the wrong outcome measures. A total of 32 studies were included for data extraction by one reviewer. Data extraction included information such as sample size, population characteristics, outcome measures, and relevant conclusions. Results are outlined in Table 3.

### 3.2. Study Characteristics 

#### 3.2.1. Location

Most studies were published in the United States (n = 9), followed by Spain (n = 4), Korea (n = 3), and Australia (n = 3). All remaining studies were published across 11 other countries, with ≤2 publications included in this review. 

#### 3.2.2. Design

Research methods of included studies were mainly non-interventional and observational (n = 31), breaking down into cross-sectional studies (n = 26), cohort studies (n = 3), and case–control studies (n = 2). One cross-sectional, one cohort, and one case–control study were retrospective (n = 3). One cross-sectional study also examined longitudinal associations. The remaining study was a pre–post study (n = 1).

#### 3.2.3. Size 

In total, 10,129 unique individuals were represented across all studies. Sample sizes ranged from 19 to 1420 individuals, with a mean of 316.53 ± 414.69 and a median of 155.5 participants in each study. In the studies that specified the sex of participants (n = 31), there were 5504 males and 4588 females. 

#### 3.2.4. Demographics

The included studies focused on children and adolescents; articles represented pediatric populations with an average from 2.04 years old to 19.95 years old. The majority of studies focused on early adolescents, aged 10–13 years old (n = 16), followed by young children aged 5–9 (n = 6), adolescents aged 14–16 years old (n = 3), toddlers aged 0–4 years old (n = 2), and young adults aged 17–20 years old (n = 1). Several studies (n = 4) included multiple age groups, three of which specified pubertal status. 

#### 3.2.5. Muscle Mass Measurements Techniques

The included articles were split in terms of MM measurement techniques: the majority used DXA (n = 21), followed by BIA (n = 7) and CT (n = 1). One study used both DXA and MRI. Two studies used other methods: US and multiple skinfold anthropometry procedure.

#### 3.2.6. Outcome Measures

Measured outcomes were divided amongst three categories: functional outcomes, including mobility, gait, activity levels, and strength (n = 13); bone outcomes, including BMC and BMD (n = 10); and other health outcomes, including cardiometabolic risk, BP, and heart rate (n = 9). 

#### 3.2.7. Participant Conditions

Studies included individuals with a range of conditions: the majority of studies focused on typically developing participants (n = 17), followed by individuals with CP (n = 7), DMD (n = 3), sarcopenia/low MM (n = 2), osteopenia/osteoporosis (n = 1), and CMD (n = 1). One study examined individuals with various neurologic disorders. 

## 4. Discussion

This systematic review demonstrates that MM measurements can correlate with a wide variety of functional, bone, and other health outcomes. While comprehensive, the discussed results are limited given the minimally relevant research relating to these topics. The outcomes discussed by the articles included in this paper can be categorized into functional/mobility outcomes, bone outcomes, and general health outcomes. The scientific literature for each of these categories is discussed here.

### 4.1. Effect of Muscle Mass on Mobility and Functional Outcomes

MM demonstrates strong relationships with mobility and functional outcomes in pediatric populations. This review found the highest number of studies (n = 13) evaluating MM correlating to measurements from the Gross Motor Function Classification System (GMFCS); the Gross Motor Function Measure (GMFM); the Ashworth spasticity scale; various jumping and walking tests; activity questionnaires; the Bruininks–Osteretsky Test of Motor Proficiency; the Spinal Alignment and Range of Motion Measure; the PEDI scale for functional ability of daily living; and measures for muscle strength, posture, and gait.

In typically developing pre-school children, LBM has been shown to be a reasonable proxy for muscle strength; because of this relationship, LBM is positively associated with and important for the determination of sit-to-stand forces [34] These relationships established in childhood are maintained in adolescence. SMM was significantly related to moderate-to-vigorous activity levels, especially for adolescents with substandard activity levels. Activity levels also showed a relationship with functional mobility and muscle strength [41]. While there is no significant difference in activity levels across sexes based on the Physical Activity Questionnaire for Children (PAQ-C), adolescent males had a significantly higher average of MM than females [39]. Given the associations between MM and functional outcomes in typically developing children and adolescents, neuromuscular conditions that affect MM and nerves would theoretically affect activity levels, muscle strength, and muscle force levels. This prediction is evidenced to be true by the literature included in this review.

Particularly in children with CP, numerous studies find that MM measurements are strongly and inversely correlated with GMFCS scores, a widely used and accepted classification of motor function [35,36,38,40]. The Modified Ashworth Scale, one of the most universally accepted clinical tools to measure muscle spasticity, demonstrates an inverse relationship with MM in children with CP [40]. The GMFM, used to measure gross motor functions, showed positive associations with MM, indicating that higher MM corresponds with higher gross motor function [32,43]. More specifically, decreased mass of the thoracic erector spinae was related to declines in lying, rolling, and sitting abilities, as measured by the GMFM. Decreased mass of the rectus abdominus and vastus lateralis was also shown to negatively impact functional abilities of daily living, including self-care activities and mobility [43].

Similar associations are observed in individuals with congenital myotonic dystrophy and DMD. In relation to comparable typically developing children, individuals with congenital myotonic dystrophy have lower measures of LBM, and this finding has been shown to correlate with lower grip/punch strengths, as measured by the Bruiniks–Osteretsky Test of Motor Proficiency [37]. In these children, LBM demonstrated associations with walking ability based on the 2 min walk test, the 6 min walk test, and the 10 m walk test; the association between MM and gait performance was found to be weaker than the grip-strength associations [37]. Similar to children with congenital myotonic dystrophy and CP, LBM was negatively correlated with functional activity in males with DMD; manual muscle testing across 32 muscle groups (in both upper and lower limbs) in boys with DMD showed a positive relationship between lean tissue mass and muscular strength [31]. In another study, the authors found that, while the association between MM and strength is strong in typically developing males, in boys with DMD, the correlation between lean MM and thigh strength was much weaker, and no correlation was found between MM and arm strength. These results suggest that weakness in DMD is likely not only attributed to lean tissue loss alone; tissue quality might also play a role and is something that could be investigated in future research [33].

Relationships between MM and functional activity are maintained beyond the discussed conditions. One study in this review included individuals with general neurological disorders (i.e., they did not discriminate between or target certain conditions): using bilateral psoas muscle area z-scores as a measurement for the degree of muscle loss/sarcopenia, researchers found a direct relationship between muscle and ability to walk; as the degree of sarcopenia increases, ambulatory function decreases [43].

### 4.2. Effect of Muscle Mass on Bone Outcomes

Bone outcomes are also correlated to MM measurements in both adults and the pediatric population. These relationships hold true for a range of bone measurements, including BMC, BMD, bone mass, bone strength, and bone thickness. 

The relationship of MM to BMC is positive and direct—higher SMM could be used to predict higher BMC in typically developing children [44,45,46,47,48]. This conclusion is rooted in Harold Frost’s mechanostat theory: biomechanical usage (i.e., muscle use) constantly affects bone strength; more mechanical usage signals a need for more bone strength, resulting in the development of more bone mass [62]. Interestingly, the relationship between MM and bone outcomes varies across sexes, pubertal stages, and ethnic backgrounds. 

As stated, LBM demonstrates a constant, positive relationship with BMC and BMD in typically developing children [46,47,52]. While these correlations exist across sexes, there are some differences between males and females. In one study, compared to their age-matched male counterparts, it was found that adolescent girls exhibit higher BMC and 67% of the difference can be explained by the variance in lean MM [44]. In another study, females had a higher BMC per unit LBM than males, even though males demonstrated higher amounts of LBM [45]. The relationship between BMC per unit LBM can vary across ethnic backgrounds. In a paper published in 2018, Heatherington-Rauth et al. found that lean MM contributes to bone structure and strength in both Hispanic and non-Hispanic females, but Hispanic females had smaller bone areas per unit LBM [50]. More studies are required to confirm these findings and further examine the differences between MM and bone characteristics in the context of sex and ethnicity. 

Another study included in this review contends that, in males, as compared with females, muscle strength might be a better indicator of bone outcomes, rather than MM [51]. BMD demonstrates a strong correlation to LBM in females [30], and sex-specific hormones are predicted to be one cause for these differences [44]. The association between LBM and BMC was absent in prepubertal females and becomes positive as they transition into puberty [46]. LBM was a strong predictor of bone mass during growth and development in males as well [49]. Bone characteristics also demonstrate relationships with muscle power output and fitness. These relationships could be explained by LBM; when LBM was not considered, no significant relationships between BMC/BMD and muscle power output or fitness were observed [44,49,52]. This was true across both sexes, and mediation of strength increases as puberty progresses. Thus, increasing LBM appears to be more important to target for therapeutic interventions to increase BMC/BMD. 

Based on this information, it would be reasonable to conclude that conditions stunting MM development in children would subsequently result in decreases in bone content and density as well. This is particularly true for females with neuromuscular disorders. Yet, this information can only be hypothesized since all included studies focus on typically developing individuals.

### 4.3. Effect of Muscle Mass on General Health and Disease Outcomes 

MM measures have the potential to be better predictors of health conditions than traditional measurements of weight or BMI. Research demonstrates that MM is related to a myriad of health indicators, including ventricular mass, BP, VO2 reserves, triglyceride (TG) levels, arterial wall function, insulin/glucose levels, high-density lipoprotein (HDL) levels, WC, and heart rate. 

MM is shown to be the best predictor for VO2 peak, a measurement of the highest amount of oxygen intake at peak exercise; VO2 peak per LBM unit is significantly stronger than using BMI [53]. It has been suggested that the weaker BMI–VO2 peak correlation is due to BMI’s inclusion of bone mass and fat mass, since bone and fat receive less than 9% of total blood flow during rest, and during exercise, this percentage decreases eve more [53]. In typically developing children, SMM specifically is highly related to cardiovascular fitness. Higher SMM resulted in a higher VO2 peak [59] and a lower percentage of VO2 reserves [60], indicating a lower perceived activity effort level; males had statistically significant higher measurements of LBM and subsequently higher VO2 peaks [53] with lower physical activity intensity-level perception despite homogenous muscle activity in box sexes [60]. Furthermore, SMM explained the variability in cardiorespiratory fitness between males and females [59].

Typically developing children with lower MM measurements were also found to have lower ventricular masses, higher heart rates, and higher BP readings [53,55,56,61]. As BP increases, vascular shear stress increases. This is consistent with findings that lower MM levels were associated with lower %flow-mediated dilation per shear rate [54]; vasculature was less responsive to accommodate high BP, which ultimately leads to a higher cardiovascular risk. 

Other studies that focused on metabolic risk examined factors such as WC, TG levels, cholesterol levels, insulin/glucose levels, and body weight. This information was then used to determine the likelihood of developing metabolic syndrome and related disorders. For typically developing children, fat-free mass (FFM) is associated with standardized BP readings [61]. The association between FFM and systolic blood pressure readings specifically was moderated by pubertal status; prepubertal children had significantly lower associations than pubertal children. This relationship was not evident between FFM and diastolic blood pressure; while an association was identified in pubertal children, there was no association identified in the prepubertal period [61]. 

Based on this information, children with lower MM measurements or children with neuromuscular conditions associated with low MM would ultimately have a higher cardiovascular and metabolic risk. These conclusions are supported by current research findings that show that children with low MM have higher BPs [55] and higher cardiovascular deterioration [56]. These children are more likely to be overweight, with larger WCs [55,56]. The blood work of these individuals indicate higher levels of TGs and insulin/glucose levels and lower levels of HDL cholesterol [55,56], which can lead to higher prevalences of metabolic syndrome [55]. In children with CP, the total energy expenditure and resting energy expenditure are lower than in able-bodied children due to limitations in physical activity. In typically developing children, FFM is associated with lower resting energy expenditure (kcal/kg/d) and higher total energy expenditure/active task. Also, the energy expenditure increases with age; this is also true for children with CP. Thus, therapeutic interventions targeting FFM increase could improve energy expenditure and utilization in these children [57]. 

## 5. Conclusions

As shown within each outcome, the research related to MM and its associations with health outcomes in a pediatric population with neuromuscular pathologies is limited and, in some areas, remains scattered. This review specifically examined populations with different conditions: some affected by neuromuscular disorders and some typically developing. This raises the distinct possibility that there is insufficient evidence and transferrable knowledge about the relationship between muscle mass and health outcomes in the intended patient population. Information and any conclusions are further limited by the inclusion of papers published only in English; this could limit the generalizability of the findings given that there may be geographic differences that are not explored by the included studies. The manuscript is also subject to publication bias, as many papers that get published report mostly positive findings. Finally, another notable limitation is the small number of reviewers that screened and extracted data. 

Regardless of these limitations, MM has significant correlations to function/mobility, bone characteristics, and general day-to-day health outcomes in both typically developing children and those with neuromuscular impairments. As a result, there is evidence to suggest that measuring and monitoring MM in children with conditions affecting MM can be indicative of these potential comorbidities and could also be used to quantify the effects of therapeutic interventions. In this context, specifically targeting an increase in MM through treatment interventions could result in functional and metabolic optimization in children with neuromuscular pathology. The fact that MM is predictive of clinical outcomes in multiple health contexts, including morbidity and mortality [2] and with the advent of affordable and reliable technology to measure it, clinically targeting MM improvement might be a worthwhile endeavor. More research is needed to confirm the findings discussed in this study and should be the basis for future work, especially for conditions that are underrepresented.

## Figures and Tables

**Table 1:** Muscle mass-related parameters and definitions.

Parameter	Unit	Definition
Fat-free mass (FFM)	kg or %	Mass of muscle, bone, tissue, and water. No fat mass included [18]
Skeletal muscle mass (SMM)	kg	Mass of skeletal muscle using BIA [18]
Lean body mass (LBM) or lean soft tissue mass	kg or %	Mass of muscle, tissue, water. No fat or bone mass included [16]
Appendicular lean soft tissue mass or appendicular skeletal muscle mass (ASM)	kg	Lean body mass (or lean soft tissue mass) without mass from head and trunk [16]

**Table 2:** Search terms and phrases.

Measurements	Conditions	Outcomes	Relationships	Populations
Muscle mass	Cerebral palsy or CP	Outcome	Association	Child
Lean mass	Quadriplegia	Gait	Relationship	Adolescent
Lean body mass	Paraplegia	Function	Correlation	Pediatric
Fat-free mass	Tetraplegia	Recovery	Predictor	
	Paralysis			

Terms in the same column, separated by rows were used with an OR Boolean. Terms separated by columns were used with an AND Boolean.

**Table 3:** Summary of findings.

Author, Year Country Design (Sample Size)	Sample Characteristics and Measurements	Outcome Measures	Conclusions
**Functional and Mobility Outcomes**			
[31] Palmieri et al., 1996 United States Cross-sectional study (n = 19)	*Individuals with DMD (n = 19):* Age, median: 11; weight (kg): 34.42 ± 14.7; LBM (%): 65.3 ± 17.7; FBM (%): 31.7 ± 17.9; functional activity score, upper body: 3.7 ± 2.2; functional activity score, lower body: 6.7 ± 3.3	DXA for total body composition Functional activity scores for muscle function Manual muscle testing for muscle strength	Total LBM was negatively correlated with both upper- and lower-extremity functional activity scores (R = −0.676, *p* = 0.021; R = −0.679, *p* = 0.002). LBM was positively correlated with manual muscle testing in 32 muscle groups (R = 0.686, *p* = 0.007).
[32] Campanozzi et al., 2007 Italy Pre–post study (n = 21)	Male = 11; female = 10 *Children with CP, improved GMFM after nutritional rehabilitation (n = 9):* Age: 6.11 ± 4.45; starting GMFM (%): 9.9 ± 6.7; Final GMFM (%): 13.7 ± 7.1; starting weight (kg): 14 ± 7.6; final weight (kg): 15.7 ± 8.3; starting FFM (kg): 12.8 ± 6.8; final FFM (kg): 13.9 ± 7; starting FBM (kg): 1.3 ± 1.1; final FBM (kg): 1.8 ± 1.4 *Children with CP, unchanged GMFM after nutritional rehabilitation (n = 5):* Age: 7 ± 3.1; starting GMFM (%): 4.14 ± 1.71; final GMFM (%): 14.3 ± 1.71; starting weight (kg): 15.5 ± 5.4; final weight (kg): 17.1 ± 6.8; starting FFM (kg): 13.7 ± 4.5; final FFM (kg): 14.1 ± 4.5; starting FBM (kg): 1.8 ± 1.6; Final FBM (kg): 2.9 ± 2	Multiple skinfold anthropometry procedure for FFM and FBM Gross Motor Function Measure for basic, gross motor function	Targeting nutritional status to significantly increase (*p* < 0.005) FFM significantly (*p* < 0.05) and positively impacts gross motor function in children with CP.
[33] Skalsky et al., 2008 United States Cross-sectional study (n = 46)	*Individuals with DMD (n = 23):* Age: 9.5 ± 2.1; lean tissue mass (kg): 19.76 ± 4.4; BMC (g): 40.44 ± 11.07; arm strength (elbow flexion + extension): 5.8 ± 2.6; thigh strength (knee flexion + extension): 15.0 ± 8.4 *Control group (n = 23):* Age: 9.3 ± 2.0; lean tissue mass (kg): 24.46 ± 7.9; BMC (g): 48.76 ± 12.33; arm strength (elbow flexion + extension): 26.6 ± 12.6; thigh strength (knee flexion + extension): 76.5 ± 42.5	DXA for lean tissue mass, fat tissue mass, and BMC Dynamometer for quantitative peak isometric strength in elbow/knee extensors and flexors	Lean tissue mass demonstrated a positive correlation with arm and thigh strength in the control group (R = 0.832, *p* < 0.001; R = 0.947, *p* < 0.001). Individuals with DMD lean tissue mass showed a weaker correlation to thigh strength and no correlation to arm strength (R = 0.514, *p* = 0.012; R = 0.164, *p* = 0.456).
[34] Hazell et al., 2014 Canada Cohort study (n = 81)	*Typically developing individuals <3 years (n = 15):* Male = 7; female = 8; age: 2.5 ± 0.4; LBM, DXA (kg): 9.2 ± 1.0; FBM, DXA (kg): 4.3 ± 1.1; sit-to-stand, relative peak force (N/kg): 11.1 ± 0.9 in males, 11.7 ± 0.9 in females; sit-to-stand, relative peak power (N/s/kg): 53.4 ± 18.5 in males, 70.4 ± 42.3 in females; jump test, relative peak force (N/kg): 20.0 ± 2.0 in males, 19.7 ± 5.0 in females; jump test, relative peak power (N/s/kg): 4.9 ± 2.5 in males, 7.6 ± 7.4 in females; body sway amplitude (mm): 1.1 ± 1.0 in males, 1.1 ± 0.4 in females; body sway velocity (mm/s): 10.1 ± 7.6 in males, 7.3 ± 3.9 in females *Typically developing individuals 3–3.9 years (n = 21):* Male = 12; female = 9; age: 3.4 ± 0.3; LBM, DXA (kg): 10.7 ± 1.5; FBM, DXA (kg): 5.1 ± 0.9; sit-to-stand, relative peak force (N/kg): 11.8 ± 1.3 in males, 10.8 ± 1.2 in females; sit-to-stand, relative peak power (N/s/kg): 76.4 ± 32.4 in males, 70.4 ± 18.2 in females; jump test, relative peak force (N/kg): 20.4 ± 2.6 in males, 18.7 ± 2.7 in females; jump test, relative peak power (N/s/kg): 6.1 ± 5.3 in males, 12.0 ± 8.1 in females; body sway amplitude (mm): 1.1 ± 0.5 in males, 0.7 ± 0.3 in females; body sway velocity (mm/s): 7.1 ± 3.9 in males, 4.3 ± 1.9 in females *Typically developing individuals 4–4.9 years (n = 28):* Male = 13; female = 15; age: 4.5 ± 0.3; LBM, DXA (kg): 12.7 ± 1.6; FBM, DXA (kg): 4.6 ± 1.3; sit-to-stand, relative peak force (N/kg): 13.4 ± 1.4 in males, 13.2 ± 1.0 in females; sit-to-stand, relative peak power (N/s/kg): 94.9 ± 39.9 in males, 94.0 ± 30.0 in females; jump test, relative peak force (N/kg): 20.3 ± 3.7 in males, 20.7 ± 3.9 in females; jump test, relative peak power (N/s/kg): 11.1 ± 6.1 in males, 18.5 ± 21.7 in females; body sway amplitude (mm): 0.8 ± 0.2 in males, 0.6 ± 0.2 in females; body sway velocity (mm/s): 5.5 ± 2.2 in males, 5.0 ± 2.6 in females *Typically developing individuals >5 years (n = 17):* Male = 10; female = 7; age: 5.3 ± 0.3; LBM, DXA (kg): 14.2 ± 2.1; FBM, DXA (kg): 4.9 ± 1.1; sit-to-stand, relative peak force (N/kg): 13.7 ± 0.6 in males, 13.2 ± 1.0 in females; sit-to-stand, relative peak power (N/s/kg): 94.8 ± 26.8 in males, 81.1 ± 21.7 in females; jump test, relative peak force (N/kg): 19.7 ± 6.3 in males, 19.6 ± 2.8 in females; jump test, relative peak power (N/s/kg): 12.3 ± 34.2 in males, 28.9 ± 9.2 in females; body sway amplitude (mm): 0.6 ± 0.2 in males, 0.7 ± 0.1 in females; body sway velocity (mm/s): 5.5 ± 5.0 in males, 8.1 ± 2.7 in females	DXA for body composition measurements Jumping test and sit-to-stand test for muscle function	LBM demonstrated importance in models to determine sit-to-stand force likely due to its indication of strength.
[35] Walker et al., 2014 Australia Cross-sectional study (n = 101)	*Individuals with CP (n = 85):* male = 58; female = 27 *GMFCS scores I and II (n = 52):* Age: 2.63 ± 0.79; FFM (kg): 11.0 ± 1.5; body fat (kg): 2.7 ± 1.4 *GMFCS score III (n = 13):* Age: 2.04 ± 0.47; FFM (kg): 9.0 ± 1.5; body fat (kg): 2.1 ± 0.8 *GMFCS scores IV and V (n = 20):* Age: 2.94 ± 0.86; FFM (kg): 9.6 ± 1.9; body fat (kg): 3.4 ± 2.0 *Typically developing individuals (n = 16):* Male = 10; female = 6; age: 3.69 ± 0.48; FFM (kg): 13.0 ± 1.4; body fat (kg): 3.9 ± 0.7	DXA for FFM and body fat measures Gross Motor Function Classification System for functioning level	Lower FFM was correlated with higher GMFCS classifications.
[36] Finbraten et al., 2015 Norway Cross-sectional study (n = 47)	*Individuals with CP:* 64 invited to participate, 52 agreed, measurements for 47 *GMFCS scores I and II:* Females = 11; males = 19; age: 13.3 ± 2.75; subscapular skinfold thickness (mm): 10.8 ± 8.2; triceps skinfold thickness (mm): 13.3 ± 6.9; body fat, DXA (%): 25.3 ± 8.5; LBM, DXA (%): 72.8 ± 9.0; bone mass, DXA (%): 3.1 ± 0.6 *GMFCS scores III, IV, and V:* Females = 7; males = 10; age: 12.83 ± 2.92; subscapular skinfold thickness (mm): 13.4 ± 6.6; triceps skinfold thickness (mm): 13.4 ± 8.0; body fat, DXA (%): 33.5 ± 7.6; LBM, DXA (%): 63.6 ± 7.2; bone mass, DXA (%): 2.9 ± 0.8	DXA for body composition Gross Motor Function Classification System for functioning level	Lower measurements of LBM were consistent with lower functional levels (i.e., higher GMFCS scores). LBM also had lower correlations to skinfold thickness (R = 0.370 for triceps, 0.382 for subscapular).
[37] Pucillo et al., 2016 United States Cross-sectional study (n = 64)	*Individuals with congenital myotonic dystrophy (n = 37):* Male = 18; female = 19; age: 7.4 ± 3.0; 10 m walk (s): 11.3 ± 6.9; 10 m run (s): 6.1 ± 3.1; time to rise from floor (s): 9.4 ± 6.3; 4 stair climb (s): 7.0 ± 7.6; 6 min walk (m): 258.3 ± 176.0; 2 min walk (m): 91 ± 58.9 *Control group (n = 27):* Male = 12; female = 15; age: 9.7 ± 2.3; 10 m walk (s): 6.8 ± 1.8; 10 m run (s): 3.2 ± 0.5; time to rise from floor (s): 2.0 ± 0.6; 4 stair climb (s): 1.5 ± 0.3; 6 min walk (m): 568.2 ± 73.2; 2 min walk (m): 193 ± 22.7;	DXA for body composition measurements Bruininks–Osteretsky Test of Motor Proficiency for fine motor control measurements	LBM is correlated with right grip strength (r = 0.84, *p* < 0.001) and right pinch strength (r = 0.76, *p* < 0.001). Correlations were less significant for the 2 min walk test (r = 0.59), 6 min walk test (r = 0.62), and 10 m walk (r = −0.38).
[38] Sung et al., 2016 Korea Retrospective case–control study (n = 146)	*Individuals with CP, GMFCS I, II, III (n = 57):* Males = 39; females = 18; age: 11.6 ± 4.5; body fat (kg): 7.6 ± 6.9; soft LBM (kg): 29.1 ± 11.8; FFM (kg): 30.6 ± 12.6; SMM (kg): 16.4 ± 7.6; BMC (kg): 1.8 ± 0.8 *Individuals with CP, GMFCS IV and V (n = 43):* Males = 25; females = 18; age: 11.4 ± 3.9; body fat (kg): 5.9 ± 5.6; soft LBM (kg): 18.4 ± 6.8; FFM (kg): 19.7 ± 7.2; SMM (kg): 9.6 ± 4.3; BMC (kg): 1.1 ± 0.5 *Typically developing children (n = 46):* Males = 24; females = 22; age: 12.8 ± 4.5; body fat (kg): 11.3 ± 7.2; soft LBM (kg): 31.8 ± 12.5; FFM (kg): 34.4 ± 13.8; SMM (kg): 18.4 ± 8.3; BMC (kg): 2.0 ± 0.8	BIA for body fat, soft LBM, FFM, SMM, and BMC measurements Gross Motor Function Classification System for functional level	Lower FFM, SMM, BMC, and soft LBM were associated with higher GMFCS scores.
[39] Wyszynska et al., 2016 Poland Cross-sectional study (n = 120)	*Typically developing primary-school children (n = 120):* Males = 59; females = 61; age: 12.09 ± 0.83; FBM (kg): 10.11 ± 5.6; MM (kg): 34.9 ± 7.45; level of physical activity (PAQ score): 2.86 ± 0.89	BIA for body composition measurements Photogrammetric method to measure posture Physical Activity Questionnaire for Children used for physical activity measurements	Individuals with higher MM showed smaller differences in scapular arrangements.
[40] Wiech et al., 2020 PolandCase–control study (n = 236)	*Individuals with CP (n = 118):* Males = 76, females = 42; age: 11 ± 3.8; GMFCS level I = 32, GMFCS level II = 56, GMFCS level III = 4, GMFCS level IV = 17, GMFCS level V = 9; Ashworth level 0 = 13, level 1 = 54, level 2 = 35, level 3 = 14, level 4 = 2; *Control individuals (n = 118):* Males = 76; females = 42; age: 11 ± 3.8	BIA for FBM, FFM, and MM measures Ashworth scale for muscle tone measurements Gross Motor Function Classification System for functional mobility level	FFM and MM are inversely related to GMFCS scores. They are also inversely related to Ashworth scores.
[41] Ito et al., 2021 Japan Cross-sectional study (n = 340)	*Typically developing children with recommended moderate-to-vigorous activity levels (n = 153):* Male = 83; female = 70; age, median: 10.0; SMM index, median (kg/m^2^): 5.98; gait speed, median (m/s): 1.20; grip strength, median (kg): 14.15; sit-to-stand test, median (5×, s): 5.94; timed up-and-go test, median (s): 7.29; one-leg standing time, median (s): 120.0 *Typically developing children with less than the recommended moderate-to-vigorous activity levels (n = 187):* Male = 82; female = 105; age, median: 9.0; SMM index, median (kg/m^2^): 5.59; gait speed, median (m/s): 1.16; grip-strength, median (kg): 12.0; sit-to-stand test, median (5×, s): 6.1; timed up-and-go test, median (s): 7.62; one-leg standing time, median (s): 93.24	Bioelectrical impedance for SMM measurements Moderate-to-vigorous physical activity questionnaire for activity measurements	There is a strong correlation between SMM and moderate-to-vigorous physical activity.
[42] Kim et al., 2023 Cross-sectional study Korea (n = 121)	*Children with neurologic disease (n = 79):* Male = 40; female = 39; ambulatory = 37, non-ambulatory = 42; age: 11.8 ± 3.7; psoas muscle area (mm^2^): 890.2 ± 481.5; psoas muscle z-score: −2.6 ± 1.1; psoas muscle index: 1.8 ± 0.9 *Children with no neurologic disease (n = 42):* Male = 16; female = 26; ambulatory = 42, non-ambulatory = 0; age: 13.1 ± 3.6; psoas muscle area (mm^2^): 1246.5 ± 754.0; psoas muscle z-score: −2.0 ± 1.2; psoas muscle index: 2.2 ± 1.1	CT was used to measure bilateral psoas muscle area to measure the degree of sarcopenia Ambulatory function was observed based on ability to walk with or without a walker	Bilateral psoas muscle area z-score (representing the degree of sarcopenia) is associated with non-ambulatory function (β = 0.436, *p* < 0.001).
[43] Masaki et al., 2023 JapanCross-sectional study (n = 32)	*Children with CP:* Male = 22; female = 10; age: 13 ± 10.8; GMFCS level I = 2; GMFCS level II = 5; GMFCS level III = 2; GMFCS level IV = 8; GMFCS level V = 15; GMFM, lying and rolling (%): 62.9 ± 31.7; GMFM, sitting (%): 47.7 ± 40.4; PEDI, self-care: 26.3 ± 24.1; PEDI, mobility: 16.8 ± 19.5; thoracic erector spinae thickness (cm): 0.81 ± 0.46; lumbar erector spinae thickness (cm): 1.83 ± 0.77; rectus abdominis thickness (cm): 0.73 ± 0.24; obliquus externus abdominus thickness (cm): 0.51 ± 0.21; gluteus maximus thickness (cm): 2.03 ± 0.64; gluteus medius thickness (cm):1.70 ± 0.74; gluteus minimus thickness (cm): 0.77 ± 0.31; rectus femoris thickness (cm): 1.27 ± 0.44; vastus intermedius thickness (cm): 0.9 ± 0.34; vastus lateralis thickness (cm): 1.25 ± 0.45; long head of bicep femoris thickness (cm): 1.41 ± 0.55; tibialis anterior thickness (cm): 1.45 ± 0.42; medial head of gastrocnemius (cm): 0.90 ± 0.27; soleus thickness (cm): 1.22 ± 0.43; spinal alignment: 6.8 ± 4.3; range of motion, lower extremities: 32.0 ± 12.3; hip flexor spasticity: 1.8 ± 0.7; hip adductor spasticity: 3.1 ± 0.7; knee flexor spasticity: 3.4 ± 0.6; plantar flexor spasticity: 3.3 ± 0.9	B-mode US device for measuring muscle thickness Spinal Alignment and Range of Motion Measure to measure spinal alignment and range of motion of lower extremities Modified Ashworth Scale for spasticity Gross Motor Function Classification System for functional mobility level Gross Motor Function Measure for gross motor function level PEDI for functional ability of daily living	Decreased MM of the thoracic erector spinae is associated with declined gross motor functions lying/rolling and sitting (R^2^ = 0.74, *p* = 0.048; R^2^ = 0.81, *p* = 0.02). Decreased MM of the rectus abdominus and vastus lateralis is associated with decreased functional abilities of daily living self-care and mobility (R^2^ = 0.76, *p* = 0.01; R^2^ = 0.90, *p* < 0.01).
**Bone Outcomes**			
[44] Vicente-Rodriguez et al., 2007 Spain Cross-sectional study (n = 278)	*Typically developing adolescents*, male = 109; female = 169; age, range: 13–18.5 years old; Raw data were included for each age group, split into years 13, 14, 15, 16, and 17–18.5 for each sex	DXA for whole-body BMC, FBM, and LBM	Differences in LBM measurements are related to differences in BMC. This is true across sexes.
[45] Dorsey et al., 2010 Cross-sectional study United States (n = 175)	*Study 1 subjects, typically developing males (n = 83):* Age: 12.0 ± 3.7; FBM, DXA (kg): 11.8 ± 9.3; BMC, DXA (kg): 2.0 ± 0.9; FFM, DXA (kg): 39.3 ± 15.3; non-bone LBM, DXA (kg): 37.3 ± 14.4; SMM, DXA (kg): 18.3 ± 8.4; SMM, MRI (kg) 18.3 ± 8.9 *Study 1 subjects, typically developing females (n = 59):* Age: 11.6 ± 3.6; FBM, DXA (kg): 15.3 ± 10.1; BMC, DXA (kg): 1.8 ± 0.6; FFM, DXA (kg): 39.3 ± 15.3; non-bone LBM, DXA (kg): 29.9 ± 8.3; SMM, DXA (kg): 14.3 ± 4.8; SMM, MRI (kg) 14.4 ± 5.1 *Study 2 subjects, typically developing males (n = 20):* Age: 8.8 ± 1.3; FBM, DXA (kg): 8.8 ± 6.3; BMC, DXA (kg): 1.2 ± 0.3; FFM, DXA (kg): 24.9 ± 4.5; non-bone LBM, DXA (kg): 23.7 ± 4.3; SMM, DXA (kg): 10.4 ± 2.7; SMM, MRI (kg) 9.9 ± 2.7 *Study 2 subjects, typically developing females (n = 13):* Age: 8.2 ± 1.0; FBM, DXA (kg): 8.5 ± 5.8; BMC, DXA (kg): 1.0 ± 0.3; FFM, DXA (kg): 20.8 ± 4.9; non-bone LBM, DXA (kg): 19.8 ± 4.7; SMM, DXA (kg): 8.6 ± 2.4; SMM, MRI (kg) 8.3 ± 2.8	DXA for BMC, non-bone LBM, skeletal mass and FBM Magnetic resonance imaging for SMM and adipose tissue	SMM measured by MRI can be used as a predictor for BMC (R2 = 0.948, *p* < 0.001). SMM measured by DXA demonstrated a weaker association (R2 = 0.929, *p* < 0.001).
[46] Wey et al., 2010 United States Cross-sectional, longitudinal study (n = 374)	*Typically developing individuals:* Males = 234; females = 140; individuals were split into the following age groups: 8–10, 10–12, 12–14, 14–16, and 16+ years old. Data were provided for each group including LBM, FBM, physical activity %, measurements at 4% radius, and measurements 20% radius. These measurements were taken again 18 months after initial tests	DXA for LBM and FBM Peripheral quantitative CT for BMC, BMD, and areaPolar stress strain index for strength	Lean MM was positively associated with cortical area (20R), bone thickness (20R), BMD (4R in males only), BMC (20R and 4R), and strength (20R and female 4R). Lean MM had no association with bone thickness (4R), BMD (20R and female 4R), and strength (4R in males only).
[47] Marwaha et al., 2016 India Cross-sectional, secondary data analysis (n = 1403)	*Typically developing adolescents:* Male = 826; female = 577; age: 13.2 ± 2.7; pubertal stage 1: male = 103; female = 39; total LBM (kg): 22.16 ± 4.21 in males, 17.61 ± 3.34 in females Pubertal stage 2: male = 194; female = 49; total LBM (kg): 27.04 ± 4.72 in males, 20.961 ± 3.02 in females Pubertal stage 3: male = 183; female = 80; total LBM (kg): 34.71 ± 6.49 in males; 25.51 ± 3.85 in females Pubertal stage 4: male = 148; female = 117; total LBM (kg): 39.71 ± 6.49 in males; 28.58 ± 4.12 in females Pubertal stage 1: male = 198; female = 292; total LBM (kg): 44.24 ± 5.59 in males, 30.38 ± 3.65 in females	DXA for LBM and bone	LBM is correlated with BMC. This correlation is strongest in the legs.
[48] Ubago-Guisado et al., 2016 SpainCross-sectional study (n = 120)	*Typically developing females:* Age: 11.32 ± 1.6 *Prepubertal females, BMI > 16.84 (n = 30):* Total BMC (g): 1230.20 ± 263.47; total BMD (g/cm^2^): 0.86 ± 0.08; total LBM (kg): 27.41 ± 5.11; total FBM (kg): 13.10 ± 4.40; percent body fat (%): 30.90 ± 6.06 *Prepubertal females, BMI ≤ 16.84 (n = 30):* Total BMC (g): 1043.03 ± 33.86; total BMD (g/cm^2^): 0.80 ± 0.07; total LBM (kg): 21.20 ± 4.12; total FBM (kg): 7.44 ± 2.35; percent body fat (%): 24.84 ± 5.27 *Pubertal females, BMI > 19.84 (n = 30):* Total BMC (g): 1911.58 ± 340.64; total BMD (g/cm^2^): 1.05 ± 0.11; total LBM (kg): 39.70 ± 3.88; total FBM (kg): 19.02 ± 4.32; percent body fat (%): 31.10 ± 4.28 *Pubertal females, BMI ≤ 19.84 (n = 30):* Total BMC (g): 1423.10 ± 271.08; total BMD (g/cm^2^): 0.92 ± 0.09; total LBM (kg): 30.52 ± 41.50; total FBM (kg): 10.04 ± 3.12; percent body fat (%): 23.58 ± 5.05	DXA for FBM, LBM, BMC, and BMD	In pubertal females, LBM is strongly correlated with BMC and BMD (r = 0.918 and r = 0.858; *p* < 0.001).
[49] Ubago-Guisado et al., 2017 Spain Cross-sectional study (n = 121)	Typically developing male participants *Swimmers (n = 41):* Age: 13.4 ± 1.0; stature (cm): 165.5 ± 9.7; LBM (kg): 41.6 ± 9.1; moderate-to-vigorous physical activity (min/day): 85.9 ± 30.4; vigorous physical activity (min/day): 11.9 ± 7.3; vertical jump (cm): 42.3 ± 6.9; standing long jump (cm): 171.0 ± 28.1; 20 m shuttle run test: 69 ± 20; Areal BMD (g/cm^2^): 0.918 ± 0.067 *Footballers (n = 37):* Age: 12.8 ± 0.9; stature (cm): 155.2 ± 9.3; LBM (kg): 35.4 ± 7.2; moderate-to-vigorous physical activity (min/day): 119.8 ± 29.7; vigorous physical activity (min/day): 22.5 ± 9.0; vertical jump (cm): 42.4 ± 6.0; standing long jump (cm): 168.7 ± 24.9; 20 m shuttle run test: 83 ± 18; areal BMD (g/cm^2^): 0.931 ± 0.071 *Cyclists (n = 29):* Age: 13.2 ± 1.0; stature (cm): 160.8 ± 9.9; LBM (kg): 37.7 ± 7.5; moderate-to-vigorous physical activity (min/day): 107.2 ± 33.3; vigorous physical activity (min/day): 18.5 ± 12.8; vertical jump (cm): 41.0 ± 6.8; standing long jump (cm): 163.5 ± 25.8; 20 m shuttle run test: 70 ± 21; areal BMD (g/cm^2^): 0.905 ± 0.086 *Nonathletes (n = 14):* Age: 12.3 ± 0.5; stature (cm): 154.5 ± 9.9; LBM (kg): 31.7 ± 5.5; moderate-to-vigorous physical activity (min/day): 83.2 ± 26.8; vigorous physical activity (min/day): 8.9 ± 4.0; vertical jump (cm): 39.5 ± 5.8; standing long jump (cm): 137.1 ± 24.5; 20 m shuttle run test: 32 ± 16; areal BMD (g/cm^2^): 0.828 ± 0.071	DXA for LBM, areal BMD, and hip structural elements Vertical and standing long jumps used for physical fitness	LBM plays a vital role in connecting fitness and bone outcomes.
[50] Heatherington-Rauth et al., 2018 United States Cross-sectional study (n = 326)	*Typically developing Hispanic females (n = 241):* Age: 10.7 ± 1.1; moderate-to-vigorous physical activity (min/day): 19.5 ± 16.0; LBM (kg): 26.0 ± 7.4; bone strength index (mg/cm^4^): 56.4 ± 22.1 *Typically developing non-Hispanic females (n = 85):* Age: 11.0 ± 1.1; moderate-to-vigorous physical activity (min/day): 23.0 ± 25.5; LBM (kg): 26.6 ± 8.6; bone strength index (mg/cm^4^): 53.1 ± 23.3	DXA for FBM and LBM Peripheral quantitative CT for bone strength	LBM was not as strong as a predictor in bone strength and bone outcomes in Hispanic females compared to non-Hispanic females.
[51] Hyde et al., 2020 Australia Cross-sectional study (n = 172)	*Typically developing males (n = 86):* Age, median: 10.93; total body (less head) BMC, median (g): 1060.39; total body (less head) BMD, median (g/cm^2^): 0.814; spine BMC, median (g): 23.6; spine BMD, median (g/cm^2^): 0.781; max handgrip strength, median (kg): 15.5; max quadriceps strength, median (N): 8.2; max hip abduction strength, median (N): 8.0; max hip flexion strength, median (N): 12.2 *Typically developing females (n = 86):* Age, median: 10.91; total body (less head) BMC, median (g): 1069.30; total body (less head) BMD, median (g/cm^2^): 0.816; spine BMC, median (g): 24.8; spine BMD, median (g/cm^2^): 0.850; max handgrip strength, median (kg): 15.0; max quadriceps strength, median (N): 7.8; max hip abduction strength, median (N): 7.7; max hip flexion strength, median (N): 11.5	DXA for BMC, BMD, and LBM measures Dynamometer for handgrip strength measures	The association between LBM and bone mass was present in both sexes but was slightly stronger in females.
[52] Rodriguez-Gomez et al., 2020 SpainCross-sectional study (n = 169)	*Typically developing prepubertal females (n = 98):* Age: 10.4 ± 1.2; body fat percentage (%): 33.5 ± 9.4; LBM (kg): 28.3 ± 5.6; whole-body BMD (g/cm^2^): 0.85 ± 0.07; femoral neck BMD (g/cm^2^): 0.72 ± 0.10; muscle output power (W/kg): 0.3 ± 0.1; CRF (mL/kg∙min): 59.4 ± 8.7 *Typically developing pubertal females (n = 71):* Age: 13.1 ± 1.6; body fat percentage (%): 38.0 ± 6.8; LBM (kg): 41.4 ± 10.0; whole-body BMD (g/cm^2^): 0.96 ± 0.10; femoral neck BMD (g/cm^2^): 0.87 ± 0.13; muscle output power (W/kg): 0.3 ± 0.1; CRF (mL/kg∙min): 52.2 ± 7.9	DXA for LBM, FBM, and BMD	LBM is a mediator between bone mass and muscle output power (Sobel test: −3.279, *p* < 0.001) and between bone mass and CRF (Sobel test: −2.691, *p* < 0.001). This effect increases with puberty.
[30] Tajaldeen et al., 2022 Saudi Arabia Cross-sectional study (n = 250)	*Control individuals (n = 123):* Male = 64; female = 59; age: 19.7 ± 1.41 males, 19.56 ± 1.23 females; fat weight (kg): 16.68 ± 5.89 in males, 33.79 ± 15.36 in females; lean weight (kg): 45.6 ± 11.03 in males, 42.88 ± 13.25 in females *Individuals with osteopenia (n = 119):* Male = 81; female = 38; age: 19.95 ± 1.31 males, 19.84 ± 1.29 females; fat weight (kg): 15.15 ± 5.42 in males, 29.52 ± 13.01 in females; lean weight (kg): 42.99 ± 8.08 in males, 39.12 ± 13.58 in females *Individuals with osteoporosis (n = 8):* Male = 5; female = 3; age: 19.4 ± 1.36 males, 18.67 ± 0.94 females; fat weight (kg): 14.82 ± 4.11 in males, 33.53 ± 3.34 in females; lean weight (kg): 37.55 ± 6.22 in males, 50.76 ± 2.85 in females	DXA was used for FBM, LBM, total fat percentages, trunk LBM, BMC, and BMD	LBM was significantly correlated with BMD in females.
**Health Outcomes**			
[53] Dencker et al., 2006 Australia Cross-sectional study (n = 248)	477 typically developing individuals invited, 248 accepted; males = 140; females = 108 *Males:* Age: 9.9 ± 0.6; end-diastolic left ventricular inner diameter (mm): 42.1 ± 3.4; total body fat, DXA (kg): 6.4 ± 5.1; LBM, DXA (kg): 26.1 ± 3.4; minutes of vigorous physical activity per day: 46 ± 20; maximal heart rate (BPM): 188 ± 16; VO2 peak (mL/min): 1423 ± 259; VO2 peak (mL/min/LBM): 54.5 ± 7.0 *Females:* Age: 9.7 ± 0.6; end-diastolic left ventricular inner diameter (mm): 40.7 ± 3.1; total body fat, DXA (kg): 8.1 ± 5.2; LBM, DXA (kg): 24.1 ± 3.5; minutes of vigorous physical activity per day: 35 ± 13; maximal heart rate (BPM): 185 ± 16; VO2 peak (mL/min): 1208 ± 203; VO2 peak (mL/min/LBM): 50.5 ± 6.9	DXA for LBM and FBM measurements Indirect calorimetry for VO2 peak	LBM was correlated with VO2 peak (r2 = 0.69, *p* < 0.05), max heart rate (r2 = 0.53, *p* < 0.001), vigorous activity per day (r2 = 0.61, *p* = 0.02), and left ventricular inner diastolic diameter (r2 = 0.60 *p* = 0.009).
[54] Hopkins et al., 2008 United Kingdom Cohort study (n = 145)	*Typically developing prepubertal individuals (n = 145):* Male = 59; female = 86; age: 10.33 ± 0.31; FBM, DXA (%): 27.1 ± 6.9; LBM, DXA (%): 70.1 ± 8.8; SBP (mmHg): 107 ± 10; DBP (mmHg): 64 ± 5; max exercise heart rate (BPM): 202 ± 26; VO2, peak (mL/kg/min): 45 ± 6; flow-mediated dilation (%): 10.1 ± 4.5	DXA for body composition measurements Actigraphy accelerometer for physical activity	There are significant correlations between LBM percentages and flow-mediated dilation percentages (r = 0.21, *p* = 0.02)
[55] Kim and Park, 2016 KoreaCross-sectional study (n = 1420)	Sex: male = 749; female = 671 *Individuals with low MM:* Age: 15.5 ± 0.2; ASM (kg): 18.0 ± 0.6; weight (kg): 67.8 ± 1.6; SBP (mmHg): 110.6 ± 1.0; DBP (mmHg): 70.2 ± 0.7; TGs (mg/dL): 162.0 ± 2.0; WC (cm): 79.4 ± 1.2; MetS prevalence: 14.8% (2.7) *Individuals without low MM:* Age: 15.6 ± 0.9; ASM (kg): 17.8 ± 0.2; weight (kg): 56.2 ± 0.4; SBP (mmHg): 107.9 ± 0.4; DBP (mmHg): 68.7 ± 0.3; TGs (mg/dL): 81.1 ± 1.6; WC (cm): 69.2 ± 0.3; MetS prevalence: 2.4% (0.5)	DXA for SMM	Having low MM was related to higher weight, higher sBP, higher dBP, higher TG levels, higher WC, and higher prevalence of MetS.
[56] Burrows et al., 2017 Chile Cross-sectional study (n = 678)	678 participants, 660 with complete data Male = 52.3%; female = 47.8%; age: 16.8 ± 0.3; obesity prevalence: 16.4%; MetS prevalence: 9.7% *Males without low MM (271):* Total LBM (%): 78.2 ± 6.0; FBM (%): 18.8 ± 5.9; WC (cm): 77.0 ± 6.5; high-density lipoprotein (mg/dL): 39.4 ± 10.2; SBP (mmHg): 113.7 ± 9.5; DBP (mmHg): 70.2 ± 6.9; TGs (mg/dL): 76.4 ± 38.3 *Males with low MM (76):* Total LBM (%): 61.2 ± 3.7; FBM (%): 35.5 ± 3.6; WC (cm): 96.3 ± 11.0; high-density lipoprotein (mg/dL): 32.9 ± 8.0; SBP (mmHg): 121.3 ± 11.3; DBP (mmHg): 73.9 ± 7.0; TGs (mg/dL): 128.8 ± 68.9 *Females without low MM (214):* Total LBM (%): 63.9 ± 5.1; FBM (%): 32.3 ± 5.2; WC (cm): 75.9 ± 8.0; high-density lipoprotein (mg/dL): 43.4 ± 11.1; SBP (mmHg): 106.2 ± 8.2; DBP (mmHg): 66.3 ± 6.4; TGs (mg/dL): 81.9 ± 41.2 *Females with low MM (99):* Total LBM (%): 51.2 ± 3.4; FBM (%): 44.9 ± 3.4; WC (cm): 92.4 ± 11.0; high-density lipoprotein (mg/dL): 40.3 ± 9.5; SBP (mmHg): 114.3 ± 10.5; DBP (mmHg): 70.3 ± 6.5; TGs (mg/dL): 100.6 ± 57.0	DXA for total FBM, total lean tissue, and ASM	Low MM is correlated with higher cardiometabolic risk. This was especially true for overweight/obese individuals.
[57] Garcia Iniguez et al., 2018 MexicoCross-sectional study (n = 79)	*Children with CP:* Age: 8y5mo ± 4y5mo; FFM, BIA (kg): 12.3 ± 5.5; FBM, BIA (kg): 4.9 ± 3.2; FFM, anthropometry (kg): 15.2 ± 6.0; FBM, anthropometry (kg): 2.1 ± 6.9; GMFCS levels I and II = 5.1%; GMFCS level III = 2.5%; GMFCS level IV = 17.7%; GMFCS level V = 69.9% *Males (n = 41):* REE (kcal/d): 911 ± 186; REE (kcal/kg/d): 53.2 ± 9.4; TEE (kcal/d): 1274 ± 261; TEE (kcal/cm/d): 11.3 ± 1.0 *Females (n = 38):* REE (kcal/d): 867 ± 177; REE (kcal/kg/d): 57.3 ± 13.7; TEE (kcal/d): 1185 ± 229; TEE (kcal/cm/d): 11 ± 1.3	BIA for FBM and FFM BIA QuadScan equations for estimations of REE and TEE	FFM measures by BIA and anthropometry are directly correlated to REE (kcal/d) and both TEE measurements (kcal/d; kcal/cm/d). FFM measures by BIA and anthropometry are negatively correlated with REE (kcal/kg/d).
[58] Summer et al., 2020 United States Retrospective cohort study (n = 1192)	*Individuals with DMD (n = 499, 2331 DXA scans)* Age, median: 11.0; whole-body LBM (g), median: 20.6Functional mobility status (FMS) level 1: n = 649 scans; FMS level 2: n = 856 scans; FMS level 3: n = 188 scans; FMS level 4: n = 71 scans; FMS level 5: n = 145 scans; FMS level 6: n = 389; FMS level 7: n = 21 scans; FMS level 8: n = 2 scans *Control group (n = 693, 3670 DXA scans)* Age, median: 13.9; whole-body LBM (g), median: 39.3	DXA for appendicular LBM National Health and Nutrition Examination Survey	Appendicular LBM and the ALM index measurements from DXA scanning can be used as biomarkers for muscle function to monitor disease progressions.
[59] Wittekind et al., 2020 United States Retrospective, cross-sectional study (n = 165)	*Typically developing children:* Age: 14.4 ± 2.5; male = 79; female = 86; total LBM (kg): 46.6 ± 12.0; LBM of right upper extremity (kg): 2.4 ± 0.9; LBM of left upper extremity (kg): 2.3 ± 0.8; LBM of trunk (kg): 20.5 ± 5.3; LBM of right lower extremity (kg): 7.2 ± 2.1; LBM of left lower extremity (kg): 7.1 ± 2.1; SMM (kg): 25.8 ± 7.3; percent body fat (%): 22.4 ± 9.6; resting exchange ratio, median: 1.24; peak absolute VO2, median (mL/min): 1914	Bioelectrical impedance for body compositional measurements Cardiopulmonary exercise testing to measure peak oxygen consumption	SMM was a stronger correlate of VO2 and CRF than total body mass.
[60] Haapala et al., 2021 FinlandCross-sectional study (n = 35)	*Typically developing males and females:* Male = 14; female = 21; age: 9.6 ± 3.0; stature (cm): 137.6 ± 9.2; SMM (kg): 14.0 ± 2.9; WC (cm): 61.1 ± 6.4; Hip circumference (cm): 74.1 ± 6.1; SMM (kg): 16.2 ± 2.4 post-pubertal adolescents, 13.1 ± 2.7 prepubertal adolescents	BIA for SMM, FBM, and FFM measures Textile electromyography for muscle activity measures	SMM showed an inverse association with VO2 reserves.
[61] Kwarteng et al., 2023 United States Cross-sectional study (n = 1405)	*Typically developing prepubertal males (n = 137):* Age: 9.7 ± 2.3; FBM (kg): 20.5 ± 13.5; FFM (kg): 27.2 ± 15.8; sBP (mmHg): 108.8 ± 12.0; dBP (mmHg): 61.9 ± 7.1; standardized sBP: 0.5 ± 1.1; standardized dBP: 0.1 ± 0.6 *Typically developing prepubertal females (n = 105):* Age: 9.3 ± 2.1; FBM (kg): 19.3 ± 9.7; FFM (kg): 27.1 ± 11.4; sBP (mmHg): 110.4 ± 12.3; dBP (mmHg): 63.2 ± 7.4; standardized sBP: 0.8 ± 1.2; standardized dBP: 0.2 ± 0.7 *Typically developing early/mid-pubertal males (n = 192):* Age: 12.7 ± 2.1; FBM (kg): 25.6 ± 18.5; FFM (kg): 44.6 ± 15.0; sBP (mmHg): 116.1 ± 12.6; dBP (mmHg): 65.0 ± 8.1; standardized sBP: 0.6 ± 1.1; standardized dBP: 0.1 ± 0.7 *Typically developing early/mid-pubertal females (n = 212):* Age: 11.6 ± 2.2; FBM (kg): 26.9 ± 13.2; FFM (kg): 37.4 ± 12.9; sBP (mmHg): 113.2 ± 12.5; dBP (mmHg): 64.3 ± 7.7; standardized sBP: 0.7 ± 1.2; standardized dBP: 0.1 ± 0.7 *Typically developing late-pubertal males (n = 157):* Age: 15.6 ± 1.4; FBM (kg): 18.3 ± 16.0; FFM (kg): 58.2 ± 11.4; sBP (mmHg): 121.7 ± 11.8; dBP (mmHg): 65.8 ± 8.0; standardized sBP: 0.5 ± 1.1; standardized dBP: −0.04 ± 0.7 *Typically developing late-pubertal females (n = 602):* Age: 15.0 ± 1.6; FBM (kg): 30.3 ± 15.2; FFM (kg): 48.6 ± 9.8; sBP (mmHg): 115.8 ± 10.8; dBP (mmHg): 65.2 ± 7.5; standardized sBP: 0.5 ± 1.0; standardized dBP: −0.04 ± 0.7	DXA for FBM and FFM Automated sphygmomanometer for BP	FFM is positively associated with standardized BP values. This relationship is mediated by pubertal stage for standardized systolic pressures.

GMFM = Gross Motor Function Measure; FFM = fat-free mass; FBM = fat body mass; DMD = Duchenne muscular dystrophy; CP = Cerebral Palsy; DXA = Dual X-Ray Absorptiometry; GMFCS = Gross Motor Classification System; PAQ = Physical Activity Questionnaire; BMC = bone mineral content; BMD = bone mineral density; LBM = lean body mass; VO2 = rate of oxygen; REE = resting energy expenditure; TEE = total energy expenditure; FMS = functional mobility status; ASM = appendicular skeletal muscle mass; US = ultrasound; sBP = systolic blood pressure; dBP = diastolic blood pressure; WC = waist circumference; MetS = metabolic syndrome; TGs = triglycerides; CRF = cardiorespiratory fitness; MM = muscle mass; BP = blood pressure.

## Data Availability

No new data were created or analyzed in this study. Data sharing is not applicable to this article.

## Supplementary Materials

The following supporting information can be downloaded at https://www.mdpi.com/article/10.3390/children11070815/s1, Table S1: checklist.
